# Caustics in the Treatment of Diseases of the Neck of the Bladder

**Published:** 1876-06

**Authors:** 


Caustics in the Treatment of Diseases of the Neck of the Uterus.—Dr. Tilt, in speaking of the efficacy of various forms of caustic in the treatment of diseases of the neck of the uterus, sums up the results of his observations in the following remarks:—1. That, in comparatively recent cases of endocervicitis, nitrate of silver, tincture of iodine, or carbolic acid suffices. 2. That chronic cases of endocervicitis had best be treated by acid nitrate of mercury or nitric acid. 3. That hyperchronic endocervicitis with considerable cervical hypertrophy requires potassa fusa cum calce, or some strong acid. He thinks Dr. Braithwaite is too general in his recommendation of nitrate of silver.—*British Medical Journal.*

